# Effects of Early vs. Late Time-Restricted Eating on Cardiometabolic Health, Inflammation, and Sleep in Overweight and Obese Women: A Study Protocol for the ChronoFast Trial

**DOI:** 10.3389/fnut.2021.765543

**Published:** 2021-11-15

**Authors:** Beeke Peters, Daniela A. Koppold-Liebscher, Bettina Schuppelius, Nico Steckhan, Andreas F. H. Pfeiffer, Achim Kramer, Andreas Michalsen, Olga Pivovarova-Ramich

**Affiliations:** ^1^Research Group Molecular Nutritional Medicine, Department of Molecular Toxicology, German Institute of Human Nutrition Potsdam-Rehbruecke, Nuthetal, Germany; ^2^Institute of Human Nutrition and Food Science, Faculty of Agriculture and Food Sciences, Christian-Albrecht-University Kiel, Kiel, Germany; ^3^Institute of Social Medicine, Epidemiology and Health Economics, Charité-Universitätsmedizin Berlin, Corporate Member of Freie Universität Berlin, Humboldt-Universität zu Berlin, Berlin Institute of Health, Berlin, Germany; ^4^Institute of Nutritional Science, University of Potsdam, Nuthetal, Germany; ^5^Digital Health-Connected Healthcare, Hasso Plattner Institute, University of Potsdam, Potsdam, Germany; ^6^Department of Endocrinology, Diabetes and Nutrition, Charité-Universitätsmedizin Berlin, Corporate Member of Freie Universität Berlin, Humboldt-Universität zu Berlin, Berlin Institute of Health, Berlin, Germany; ^7^German Center for Diabetes Research (DZD), Neuherberg, Germany; ^8^Laboratory of Chronobiology, Institute for Medical Immunology, Charité-Universitätsmedizin Berlin, Corporate Member of Freie Universität Berlin, Humboldt-Universität zu Berlin, Berlin Institute of Health, Berlin, Germany; ^9^Department of Internal and Integrative Medicine, Immanuel Hospital Berlin, Berlin, Germany

**Keywords:** time-restricted eating, meal timing, circadian clock, obesity, diabetes, metabolism, inflammation, sleep

## Abstract

**Background:** Time-restricted eating is a promising dietary strategy for weight loss, glucose and lipid metabolism improvements, and overall well-being. However, human studies demonstrated contradictory results for the restriction of food intake to the beginning (early TRE, eTRE) or to the end of the day (late TRE, lTRE) suggesting that more carefully controlled studies are needed.

**Objective:** The aim of the ChronoFast trial study is to determine whether eTRE or lTRE is a better dietary approach to improve cardiometabolic health upon minimized calorie deficits and nearly stable body weight.

**Methods:** Here, we present the study protocol of the randomized cross-over ChronoFast clinical trial comparing effects of 2 week eTRE (8:00 to 16:00 h) and lTRE (13:00 to 21:00 h) on insulin sensitivity and other glycemic traits, blood lipids, inflammation, and sleep quality in 30 women with overweight or obesity and increased risk of type 2 diabetes. To ensure timely compliance and unchanged dietary composition, and to minimize possible calorie deficits, real-time monitoring of dietary intake and body weight using a smartphone application, and extensive nutritional counseling are performed. Continuous glucose monitoring, oral glucose tolerance test, 24 h activity tracking, questionnaires, and gene expression analysis in adipose tissue and blood monocytes will be used for assessment of study outcomes.

**Discussion:** The trial will determine whether eTRE or lTRE is more effective to improve cardiometabolic health, elucidate underlying mechanisms, and contribute to the development of recommendations for medical practice and the wider population.

**Clinical Trial Registration:** www.ClinicalTrials.gov, Identifier [NCT04351672]

## Introduction

Obesity and associated diseases, such as type 2 diabetes, are a rapidly expanding problem of our society and a substantial burden on healthcare systems. The success of currently recommended lifestyle interventions (i.e., dietary modifications and increased physical activity) as a first-choice obesity treatment is limited (1, 2). Novel effective approaches to lose weight and improve glycemic control are urgently needed. Latest research revealed that not only the quality and quantity of food, but also the daytime and duration of food consumption are important factors for metabolic regulation (3–5). This phenomenon is based on the tight interaction of endogenous circadian clock and metabolism.

The circadian clock is an endogenous timing system generating approximately 24-h long rhythms of physiology, metabolism, and behavior to align them to the day-night changes and corresponding sleep/wake and fasting/feeding phases (6, 7). Accordingly, a large part of components of carbohydrate, protein, cholesterol, lipid metabolism, and mitochondrial function displays circadian oscillations (6, 8–12), whereas genetic clock disruption leads to metabolic disturbances (13–15). Secretion and action of metabolic hormones, e.g., cortisol, melatonin, adiponectin, leptin, resistin, ghrelin, and cytokines, also undergo circadian regulation (16–20). In particular, synthesis, and secretion and sensitivity to insulin are controlled by the circadian clock (19, 21). Notably, metabolic disturbances induced by nutrient imbalance or excess, such as type 2 diabetes, obesity, and metabolic syndrome, are associated with blunted or changed circadian rhythms (10, 22–24).

Proper functioning of circadian clocks and its interaction with metabolism are critical for maintaining metabolic health. The experimental misalignment between endogenous clock rhythms and rhythms of food intake and sleep/wake induces weight gain and metabolic disruptions in rodents (15) as well as dysregulation of the circadian transcriptome, glucose intolerance, and changes in lipid metabolism and metabolome in experimental human studies (18, 19, 25–29). Shift work and chronic jet lag are associated with an increased risk of obesity, type 2 diabetes, and cardiovascular diseases in epidemiological human studies (16, 17). In addition, the increasingly frequent late lifestyle and related “social jet lag”, which describes the discrepancy between an early sleep/wake and early eating schedule during work days and a later schedule during days off, is associated with a disruption of circadian rhythms (4, 7, 26).

Furthermore, the timing of food intake during the course of the day can also affect body weight regulation and metabolic health. Indeed, an evening meal induces higher postprandial glucose concentrations and different secretion of insulin and incretins compared with the same meal consumed in the morning (21, 30, 31). Glucose tolerance, skeletal muscle fatty acid oxidation, and diet-induced thermogenesis are higher in the morning than in the evening, at least in healthy humans (7, 32). These findings suggest that eating earlier in the daytime might be more optimal for food consumption, whereas delayed eating rather leads to metabolic dysfunction (33). Moreover, higher evening caloric intake has been found to predispose to greater total daily caloric intake, obesity, and metabolic diseases (34, 35), although some studies did not confirm this finding (36, 37). In addition, earlier lunch, consumption of largest meal of the day for breakfast, or scheduling of all meals earlier in the day seems to improve weight loss success, glycemic control, and blood lipids (38–40).

Even though early eating appears to be metabolically beneficial, eating patterns in our modern society are rather different. More than half of American and Indian adults spread their daily caloric intake over 15 h or longer, consuming a larger portion of the daily energy intake (30–45 %) toward the late afternoon and evening hours (41–43). This supported the idea that shortening of eating period might have beneficial effects on cardiometabolic state and weight loss (41, 42, 44). Therefore, time-restricted eating (TRE), a dietary approach limiting daily eating window to usually under 10 h/day attracted increasing attention in media and research. In mice, time-restricted feeding increases the amplitude of circadian clock rhythms and is protective against high-fat diet (HFD)-induced obesity, glucose intolerance, leptin resistance, hepatic steatosis, and tissue inflammation compared with *ad libitum* HFD feeding (45, 46). Human studies on TRE characterized it as a well-tolerated and promising strategy for weight loss and improvement of metabolic outcomes for the prevention and therapy of obesity and type 2 diabetes in medical practice and the general population. Notably, TRE is an easy to use dietary approach, because it does not require extensive nutritional knowledge and control of food quantity and quality. Most human TRE studies reported modest reduction of body weight (41, 47–59), fat mass (47, 49, 51, 52, 54, 56, 57, 59), and waist circumference (53, 54). Further beneficial effects of TRE are elevated adiponectin levels (52, 56), decreased levels of inflammatory (52) and oxidative stress (51, 60) markers, lowered blood pressure (50, 54, 56, 60), and even improvement in sleep quality and quality of life (41, 53). TRE has also shown to improve fasting glucose and postprandial glucose levels (49, 52, 59, 61), mean daily glucose (58, 61), and insulin resistance (51, 52, 55, 60, 61), as well as blood triglyceride (47, 49, 52, 58, 62), total cholesterol, and LDL cholesterol levels (54, 62). However, the observed effects of TRE are highly variable and particularly contradictory, especially concerning metabolic outcomes. Indeed, some TRE studies demonstrated no alteration or worsening of glucose (47, 48, 50, 51, 56, 57, 63) and lipid metabolism markers (48, 50, 51, 53–57, 60, 61, 63).

This inconsistency might be caused by the wide spectrum of investigated TRE regimens on human trials. This might be explained by different study designs, such as eating window duration and daytime, changes in calorie intake, duration of intervention as well as by study subject cohorts different in metabolic status, age, gender, chronotype etc. Most studies were short-term trials with interventions lasting from 4 days to 16 weeks and relatively small sample sizes, with only several trials involving more than 50 participants (47, 48, 62, 64). Daily mealtimes vary from 4 to 12 hours, and food intake windows vary from early (eTRE) to late in the day (lTRE). To date, it remains unclear whether there are significant differences between early- and late-TRE based on current knowledge of circadian biology and nutrient metabolism. Until now, only one published study directly compared eTRE and lTRE in a cross-over design (58) and found no significant difference in the improvement of postprandial glucose and fasting triglycerides between two dietary patterns, which can be explained by manifested weight loss after both treatments.

Notably, most human TRE studies published, so far, did not carefully monitor calorie intake. Most published trials reported a reduction in energy intake, because individuals mostly reduce their caloric intake spontaneously when the time window for eating is restricted (41, 51, 54, 55, 62, 65). This raises the question whether beneficial metabolic effects of TRE are induced by the shortening of the eating window or by calorie restriction (and respective weight loss) alone. Furthermore, most published human TRE studies did not carefully monitor dietary macronutrient content, which could lead to false data interpretation, e.g., if subjects have to skip high-fat or sweet snacks or alcohol drinks often consumed in the evening. Third, the level of physical activity before and during the intervention was not carefully assessed in studies so far, which may induce a potential bias. Finally, physiological and molecular mechanisms induced by eTRE and lTRE in humans have not been investigated and compared yet.

Therefore, the aim of our study will be to compare cardiometabolic effects of eTRE vs. lTRE in a cross-over study with careful control of timely compliance, dietary composition, calorie intake, and physical activity to minimize a possible calorie deficit or even achieve stable body weight. The study will assess effects of 8-h eTRE and lTRE on blood glucose and lipid levels, metabolic hormones related to glucose metabolism, hormones of appetite regulation, adipokines and inflammatory markers, blood pressure, satiety and hunger scores, sleep quality, and social and economic decision behaviors to determine whether eTRE or lTRE is a better dietary approach for the improvement of cardiometabolic health. The trial will be conducted in women with overweight or obesity who have an increased risk of type 2 diabetes and other obesity-associated metabolic disturbances that can potentially be reversed by lifestyle changes, e.g., by change in meal timing. The trial will give further insights into the effects of eTRE in comparison with lTRE and contribute to the recommendation development for the use of the TRE approach in medical practice and general population.

## Materials and Methods

### Study Design

**Figure 1:**
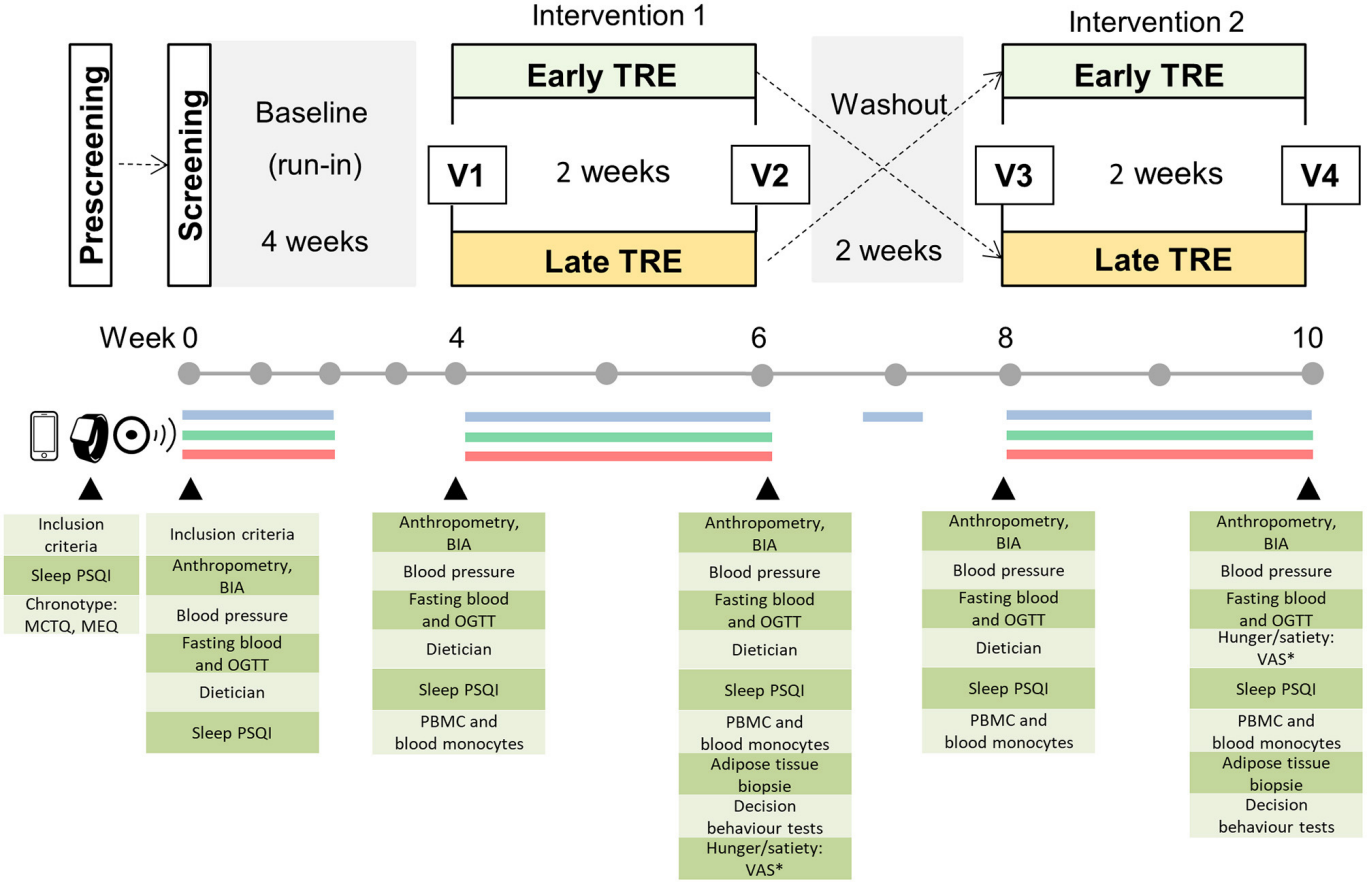
Study design. Prescreening of potential participants is performed by phone and mail, and enrolled subjects are invited to the clinical research center for a screening examination. The cross-over study includes a 4-week baseline (run-in) and two 2-week dietary intervention periods separated by a 2-week washout phase. Blue line: assessment of eating pattern using a smartphone app or paper-based handwritten food and body weight diaries; green line: continuous glucose monitoring, red line: assessment of activity and sleep patterns by actigraphy and sleep diary for 14 consecutive days during the run-in (baseline period) and each intervention. In the washout phase, food intake monitoring is performed for 3 consecutive days. ^*^ Visual Analog Scale (VAS) is assessed at 8:00 and 20:00 h on the day before visit 2 and visit 4; V, visit at the examination center.

The study named “ChronoFast” is a 10 week randomized, controlled cross-over clinical trial that will be performed in a German population of female subjects with overweight or obesity. The study will consist of two 2 week dietary intervention periods: (1) early TRE and (2) late TRE. After a 4 week long run-in period, the participants will be randomly assigned to either 2 weeks of eTRE or 2 weeks of lTRE as their first intervention. The first phase of intervention will be separated by a 2 week washout period from the second phase of TRE intervention that will be the opposite of the first intervention (Figure 1). The duration of the run-in phase, and the washout and intervention phases were chosen to exclude the effect of monthly ovarian cycle in women (both intervention phases will be approximately at the same phase during both interventions). Previous data of our and other groups show that change in dietary pattern for 2 weeks is sufficient to induce strong changes in metabolic state (55, 58, 66). Furthermore, the short 2 week duration of TRE interventions will make the rigorous monitoring of food intake and physical activity more feasible and will allow for the achievement of minimal variation in body weight.

During the run-in period, the participants will be instructed to consume their habitual food at the usual day time. In the early TRE (eTRE) intervention, the study participants will be asked to consume their habitual food (and the habitual daily amount of food) between 8:00 and 16:00 h. In the late TRE (lTRE), the study participants will consume their usual food between 13:00 and 21:00 h. Both dietary regimens will result in an 8-h eating period and 16-h fasting period during the 24 h day. During the 2 week washout phase, the participants will be asked to return to their habitual eating window. During the whole study, the participants shall avoid alcohol consumption and maintain their usual lifestyles, which include regular physical activity and sleep patterns.

Eating behavior will be assessed using a smartphone app or paper-based handwritten food diaries to determine the macronutrient composition, energy intakes, and timely compliance for 14 consecutive days during the run-in (baseline period) and each intervention. For these 14 days during each study period, the participants will be simultaneously fitted with a continuous glucose monitoring (CGM, FreeStyle Libre 2; Abbott, Chicago, IL, USA) device to measure interstitial glucose, whereas activity and sleep patterns will be tracked by an accelerometer (ActiGraph wGT3X-BT; ActiGraph, Pensacola, FL, USA) (Figure 1). Furthermore, the participants will be advised to maintain their habitual sleep–wake routine over the course of the study and complete sleep diaries and weight protocols for 14 days during the run-in phase and both intervention periods. On the last day of both intervention periods, the subjects will also complete the Visual Analog Scale to assess satiety and hunger scores.

The study includes a screening examination before the run-in phase and four visits before and after each intervention periods (Figure 1). On these days, the participants will visit the examination center for anthropometric measurements (body weight and composition, waist and hip circumferences), assessment of blood pressure, collection of fasting blood samples for routine laboratory tests, and metabolic biomarker analysis as well as a 3-h oral glucose tolerance test (OGTT) for the additional assessment of glycemic control (Figure 1). At visits 2 and 4, subcutaneous adipose tissue (SAT) samples will be additionally collected. A detailed overview of visits and assessments is shown in Table 1.

**Table 1:** Overview of visits and assessments.

**Method/procedure**	**Main parameters**	**Pre-screening (phone, mail)**	**Screening**	**Visit 1**	**Visit 2**	**Visit 3**	**Visit 4**
Week			0	4	6	8	10
Inclusion/exclusion criteria		•	•				
Chronotype questionnaires: MCTQ, MEQ	Chronotype	•					
Physical examination			•				
Informed consent			•				
Randomization			•				
Anthropometric measurements	Body weight, waist and hip circumferences		•	•	•	•	•
Bioelectrical impedance analysis (BIA)	Fat and lean mass and percentage		•	•	•	•	•
Blood pressure measurement	Blood pressure		•	•	•	•	•
Fasting blood sample	Clinical biochemistry, other metabolic and inflammatory biomarkers		•	•	•	•	•
Oral glucose tolerance test	Glucose, insulin, GIP, GLP-1		•	•	•	•	•
PBMC and blood monocyte sample	Inflammatory gene expression, BodyTime assay			•	•	•	•
Adipose tissue biopsie	RNA-Seq and pathway analysis				•		•
PSQI sleep questionnaire	Subjective sleep quality	•	•	•	•	•	•
Decision behavior tests	Social and economic decision behavior				•		•
Satiety and hunger scores (VAS)^a^	Satiety and hunger				•		•
Dietician / Food diary training^b^			•	•	•	•	
ActiGraph fixation^2^	Physical activity, objective sleep quality		•	•		•	
CGM sensor fixation^2^	Glycemic variability and response to the food intake		•	•		•	

a *at 8:00 and 20:00 h on the day before visit 2 and visit 4*.

b *data collection for 14 consecutive days. •presents the implementation of the listed procedure at a given time, i.e. Pre-screening, Screening or visit 1–4*.

The study will be conducted in accordance with the Helsinki Declaration of 1975. Study protocol was reviewed and approved by the Ethical Committee of the University of Potsdam, Germany (EA No. 8/2019). The nature, benefits, and risks of the study were explained to all the subjects, and their written informed consent were obtained prior to participation. The study was conducted in the outpatient department of the German Institute of Human Nutrition by the research group Molecular Nutritional Medicine, with support from the staff of Human Study Center. The study was registered at www.clinicaltrials.gov under the identifier NCT04351672 on April 17, 2020.

### Subjects and Eligibility

The study was performed on 30 female individuals with overweight or obesity. This is a hallmark of the study, because only few published trials were focused on female subjects (67, 68). Inclusion criteria were BMI 25–35 kg/m^2^ and age 18–70 years. Exclusion criteria were prior diagnosis of diabetes, weight changes > 5% within the last 3 months, current shift work or history of shift work, travel across more than one time zone a month before the study or during the study period, poor quality of sleep (Pittsburgh Sleep Quality Index (PSQI) score > 10), eating disorders, severe internal or psychiatric disorders, and other conditions that might influence the outcome of the study, as indicated in Table 2.

**Table 2:** Inclusion and exclusion criteria.

**Inclusion criteria**
• Women • Age 18–70 years • BMI 25–35 kg/m^2^
**Exclusion criteria**
• Shift work • Travel across more than one time zone one month before a study or during the study period • Weight changes of more than 5% in the last 3 months • Pregnancyor breastfeeding • Severe intestinal disease, bariatric surgery in the past • Food allergies (individual inclusion possible after consultation with the doctor) • Currentspecialdiets • Poor quality of sleep (PSQI score < 10) • Diabetes type 1 or type 2 • Severekidneydisease • Severeliverdisease • Cardiac infarction or stroke in the last 6 months • Cancer cases in the last 2 years • Glucocorticoid therapy (oral) • Coagulationdisorders • Taking anticoagulant medication (inclusion if pausing medication is possible) • Severeanaemia • Systemicinfections • Severe psychiatric illnesses, addictions or depression • Other diseases/surgeries/medications affecting glucose metabolism, appetite or immune function

### Screening and Recruitment

The study participants were recruited from the Berlin-Brandenburg area, Germany, by means of personal interview, flyers and posters in public places, and advertising through newspapers and classified ads websites. Initial screening with interested women was performed by phone to provide detailed information about the study, determine the eligibility of potential participants, and check their interest in the study. Following the telephone screening, the potential subjects were given chronotype questionnaires, a PSQI sleep quality questionnaire, and informed consent form by mail and were asked to return them back. Subjects who scored < 10 on the PSQI and fulfilled all the inclusion criteria up to that point were invited to the clinical research center at the German Institute of Human Nutrition for a screening examination.

The screening examination of participants included anthropometric measurements, fasting blood sampling, OGTT, interviews on lifestyle and medical history, and standard physical examination by the study physician. They met a dietician for behavioral nutritional consulting who gave instructions about the documentation of food intake as described below. Furthermore, a CGM sensor and ActiGraph were fixed on the body of the participants, and they received instructions for usage of those devices.

### Run-In Phase

During the first 2 weeks within a 4 week run-in phase, the participants were asked to document their food selection, consumed amount, and time of each meal daily using a smartphone app or handwritten diary to determine their habitual eating pattern. The participants were informed about the importance of maintaining constant body weight for the whole duration of the study. They were instructed to record their weight daily and report any weight fluctuation of ≥ 700 g on 2 consecutive days. During the first 2 weeks of the run-in phase, the study subjects wore a CGM sensor and an ActiGraph device. Furthermore, they wrote sleep diaries and weight protocols.

### eTRE and lTRE Interventions

Before and after each intervention period (eTRE and lTRE), the participants visited the examination center where anthropometrical measurements, fasting blood sampling, and OGTT were performed after overnight fast, and PSQI sleep questionnaire were completed (visits 1–4, Figure 1). Subcutaneous adipose tissue (SAT) biopsies were taken after both intervention periods (visits 2 and 4). At visits 1 and 3, the CGM sensor and ActiGraph were fixed on the body, and nutritional consulting was performed by the dietician who explained eating behavior during the TRE intervention.

The participants were instructed to consume their habitual food between 8:00 and 16:00 h during the early TRE intervention phase and between 13:00 and 21:00 h during the late TRE intervention period to achieve a daily eating window of 8 h and fasting window of 16 h (Figure 1). The subjects were asked to consume their usual kind and amount of food within the 8 h eating window but were free to divide their food intake into as many meals or snacks as desired. For guidance, the participants received a copy of their individual food records and daily caloric intakes made in the run-in phase. Outside the eating window, the participants were allowed to consume water and non-caloric drinks e.g., tea and black coffee without sugar and milk, and, in limited amounts, very low-caloric diet sodas, mints, or chewing gum with sweeteners as a tool that may increase compliance. Telephone calls in the middle of each intervention and the run-in phase (7 days after visiting the examination center) were conducted in order to promote adherence to the assigned program and answer questions. Again, for the 14 consecutive days of the TRE intervention, the subjects documented eating behavior, wore the CGM sensor and ActiGraph device, and completed sleep diaries and weight protocols (Figure 1; Table 1).

### Washout Phase

During the 2 week washout phase, the participants were asked to return to their habitual eating window before the beginning of the study and document their food consumption for 3 consecutive days (2 weekdays and 1 weekend day).

### Measurements

#### Anthropometric Measurements, Body Composition, and Blood Pressure Assessment

Anthropometric measurements were performed at the screening examination and visits 1–4 following overnight fast directly after the arrival of participants in the examination center. Body weight and height were measured with a digital scale and a wall-mounted stadiometer, respectively. Waist and hip circumferences were measured with a measuring tape. Body composition was measured using a bioelectrical impedance analysis (BIA) device (Bioimpedance Analyzer Quantum S; Akern, Italy) and fat and lean mass (kg and percentage) were calculated with the BodygramTM software (Akern).

Blood pressure was measured during every visit manually on the left upper arm after at least a 3-min rest. The measurement was performed three times, and then the values were averaged.

#### Eating Behavior Documentation and Analysis

The subjects documented all their caloric intakes (food selection, amount and time of each meal) for 14 consecutive days during the run-in period, during both TRE intervention phases, and for 3 consecutive days in the washout phase. The participants documented their food intakes either in form of paper-based handwritten food diaries or using the Fddb Extender app that is a free smartphone application to keep an electronic food diary (https://fddb.info/) (69). The study subjects were instructed to weigh all food whenever possible and to supply information on food details (e.g., brand names) and mealtimes. When weighing was not possible (e.g., when dining out), they were instructed to record their food intake in household measures (cups, glasses, teaspoon, etc.). Subjects who document their nutrition with the Fddb extender app obtained detailed introduction on how to use the app at their screening visit. Subjects who were not familiar with using a smartphone completed paper-based handwritten dietary records, which were later incorporated in the Fddb app by a study assistant.

Energy intake and diet composition were assessed using the free FDDB food database (Fddb Internetportale GmbH, https://fddb.info/) for both the paper- and app-recorded intake to ensure comparability. Dietary records from each condition were analyzed for total energy intake and macronutrient composition. The reported time of all food intakes was used to monitor participant adherence to the designated eating window. To determine the daily eating window, the time interval between the first and last caloric intake of the day was calculated. These self-reported time windows were verified by examining the pattern of glucose excursions using the 24-h CGM data.

At the end of the study, each participant received individual nutritional counseling with an aim to reduce body weight based on the recommendations of the German Nutrition Society (DGE) guidelines and adjusted to their eating behavior and health condition (e.g., pre-diabetes).

#### Continuous Glucose Monitoring

The study participants were fitted with a Freestyle Libre 2 CGM system (Abbott) during the run-in period and during both dietary intervention phases. CGM measured interstitial fluid glucose every 15 min for 14 consecutive days, using a subcutaneous sensor placed in the upper arm area. Each participant was educated on how to wirelessly scan the Freestyle Libre 2 (Abbott) sensor with the corresponding FreeStyle Libre 2 (Abbott) portable reader. At the next examination visit, the glucose data were saved in the study database. Mean daily glucose level, mean amplitude of glycemic excursions (MAGEs), mean of daily differences (MODDs), and continuous overall net glycemic action (CONGA) were analyzed (70).

#### OGTT and Blood Sample Analyses

At screening examination and visits 1–4, an OGTT was performed starting at 09:30 h after overnight fast. Following the insertion of an intravenous catheter and collection of fasting blood sample (−5 min), the participants ingested a drink containing a 75 g load of glucose within a 5 min time frame. Blood samples were drawn at 30, 60, 90, 120 and 180 min of the test using EDTA-containing and serum monovettes (Sarstedt, Germany). For the analysis of incretins, 10 μg/ml aprotinin (Roth, Germany) and 50 μM DPP4 inhibitor (Merck Millipore, Darmstadt, Germany) were additionally added to the blood samples. EDTA monovettes were centrifuged directly, whereas the serum monovette was kept for 10 min at room temperature to clot before centrifugation at 1,800 × g for 10 min at 4°C. The serum and plasma samples were stored at −80°C until analysis.

Measurement of routine laboratory parameters, such as aspartate aminotransferase (ASAT), alanine aminotransferase (ALAT), gamma-glutamyl transferase (GGT), creatinine, urea, uric acid, C-reactive protein (CRP), glucose, glycated hemoglobin (HbA1c), and blood lipids (total cholesterol, high density lipoprotein (HDL)-cholesterol, triglycerides (TG), and non-esterified fatty acids (NEFAs) were performed using ABX Pentra (Horiba). LDL cholesterol was determined using the Friedewald equation (71).

Measurement of metabolic and inflammatory biomarkers in the plasma and serum samples was performed by commercial Enzyme-linked Immunosorbent Assays (ELISA) or multiplex assays according to the recommendations of the manufacturer. Biomarkers included but were not limited to hormones related to glucose metabolism (insulin, C-peptide, glucagon, glucagon-like peptide-1, and glucose-dependent insulinotropic polypeptide), hormones of appetite regulation (peptide YY and ghrelin), adipokines (adiponectin and leptin), and inflammatory markers (interleukine 6, tumor necrosis factor alpha, and monocyte chemoattractant protein 1).

#### Subcutaneous Adipose Tissue Biopsies and Blood Monocyte Collection

Subcutaneous adipose tissue biopsies were collected at visits 2 and 4 at the level of the umbilicus using a well-established technique (72). The skin was anesthetized with 1% lidocaine. A small incision was made, and 1 g of adipose tissue was removed under sterile conditions using a cutting needle biopsy handy (14 G Somatex™, Germany). SAT biopsies were collected immediately before OGTT and frozen in liquid nitrogen until further analyses. An RNA-Seq analysis of the SAT samples was performed to elucidate and compare signaling pathways affected in the adipose tissue by eTRE and lTRE and validated by quantitative real-time PCR.

Peripheral blood mononuclear cells were isolated from fasting EDTA blood using BD Vacutainer® CPTTM with an integrated FICOLL™-gradient (BD Biosciences, East Rutherford, NJ, USA). Blood monocytes were isolated from the peripheral blood mononuclear cell (PBMC) samples using whole-blood CD14 microbeads (Miltenyi Biotec, North Rhine-Westphalia, Germany) as described (73). The PBMC and monocyte samples were stored at −80°C until the analysis of inflammatory gene expression and BodyTime assay for the determination of TRE effects on internal circadian time (74).

#### Actigraphy and Physical Activity Assessment

Twenty-four-hour physical activity was measured with the ActiGraph wGT3X-BT (ActiGraph) activity monitor to ensure that it will stay constant for the whole duration of the study. The participants wore the accelerometer on the wrist of their non-dominant arm for 14 days during the run-in phase and during both TRE interventions simultaneously with CGM and nutrition assessment. The subjects were asked to wear the ActiGraph device day and night and only take it off if taking a bath or swimming. The device contains a wear time sensor to simplify compliance monitoring. Furthermore, the participants documented the times they were not wearing the activity monitor. Individual physical activity levels, energy expenditure, and metabolic equivalent of task (MET) were analyzed with the ActiLife software version 6.13.4 (ActiGraph).

#### Sleep Analysis

Because subjects reported sleep improvement upon the shortening of eating time in the study of Gill et al. (41), we analyzed TRE effects on sleep quality and duration using subjective and objective methods. During the course of each examination day, the participants assessed their sleep quality during the previous 2 weeks by the Pittsburgh Sleep Quality Index (PSQI) (75), which evaluates seven components of sleep architecture, subjective sleep quality, sleep latency, sleep duration, habitual sleep efficiency, sleep disturbances, use of sleeping medication, and daytime dysfunction. The objective analysis of sleep was performed using ActiGraph wGT3X-BT devices following the assessment of sleep latency, total sleep time, sleep efficiency, and sleep fragmentation index by the ActiLife software as described (76). Sleep timing for this analysis was obtained from the sleep diaries where the subjects documented their bedtime and wake-up time for 14 days during the wearing of ActiGraph device, and added manually.

#### Questionnaires and Decision Behavior Tests

The chronotypes of the subjects were assessed using the Munich Chronotype Questionnaire (MCTQ) and the Horne-ÖstbergMorningness-Eveningness Questionnaire (MEQ).

Satiety and hunger scores were assessed using Visual Analog Scale (VAS) for (1) desire to eat, (2) hunger, (3) satiety, and (4) capacity to eat, as described previously (21). VAS surveys were completed once in the morning at 8:00 h and once in the evening at 20:00 h on the last day of each intervention (the day before visits 2 and 4).

Social and economic decision behaviors were assessed by computer tests together with the Barratt Impulsiveness Scale and the UCLA Loneliness Scale in cooperation with Prof. Soyoung Park (Dept. Decision Neuroscience and Nutrition, German Institute of Human Nutrition).

### Primary and Secondary Outcomes

The primary outcome was the insulin sensitivity assessed using Matsuda index in OGTT (77). Secondary outcomes included body weight, BMI and body fat content, fasting glucose, homeostasis model assessment of insulin resistance (HOMA-IR), indices of insulin secretion and beta-cell function assessed in OGTT, mean daily glucose and daily glycemic variation indices assessed in CGM, levels of hormones related to glucose metabolism, hormones of appetite regulation, adipokines and inflammatory markers, blood lipid and liver enzyme levels, systolic and diastolic blood pressures, satiety and hunger scores, sleep quality and duration, physical activity, social and economic decision behaviors, dietary calorie intake, composition, timing, and eating window duration. The main exploratory outcome was the activity of signaling pathways and expression of metabolic and inflammatory genes in adipose tissue and blood monocytes.

### Statistical Analysis

Power calculation was completed using the G-Power software v.3.1 (78) for the primary end point change in insulin sensitivity. The power calculation was based on the difference in insulin sensitivity in the meal tolerance tests conducted in the morning and in the evening in subjects with IFG/IGT in our previous study (21). For the sample size of 30 study participants, the planned study was powered to detect an effect size of 0.53 at a significance level of 0.05 and statistical power of 80%. The allocation of subjects to study arms was performed by matching for age and BMI using a minimization method and the MinimPy software (79).

Statistical analyses were performed with the SPSS 20.0 software (SPSS, Chicago, IL, United States). To estimate the effects of the dietary interventions on anthropometrical and metabolic parameters, linear mixed-effect models were applied with treatment, period, and residual effect as fixed factors and the study participants as a random factor. Sampling distribution was analyzed by Shapiro–Wilk test. Not normally distributed data were log-transformed before analysis. For NEFA, glucose, and meal-induced hormone secretion, areas under the curve (AUC) and incremental AUC (after subtraction of the baseline area, iAUC) were determined by trapezoidal method. Insulin sensitivity and insulin secretion indices in OGTT were calculated as described (80). For the comparison of two groups, paired Student's *t*-test or Wilcoxon test was performed depending on the distribution of data. A *p*-value < 0.05 was considered statistically significant. For multiple testing, false discovery rate correction was applied.

### Data Handling

All data collected in the applied project were stored in the database on servers of the German Institute of Human Nutrition and backed up regularly. Applicant access to these databases is guaranteed. During the process of data collection, the study data were pseudonymized, and the identifying data were stored separately from the research data. After completion of data collection, the data were anonymized for the process of research. In accordance with the rules of good scientific practice, the anonymized data will be archived on servers of the German Institute of Human Nutrition infrastructure for at least 10 years.

## Results

The ChronoFast clinical trial is currently ongoing. The estimated primary completion date is December 2022. The trial aims to compare effects of eTRE and lTRE on insulin sensitivity, other cardiometabolic and inflammatory markers, and sleep quality when calorie deficits are minimized and body weight is nearly stable. Further objective is to elucidate molecular mechanisms underlying metabolic changes in adipose tissue and blood monocytes in women with overweight and obesity.

### Expected Findings

The study hypothesis is that both interventions will improve glycemic control, lipid metabolism, inflammation, and sleep quality because of reduced eating time while minimizing weight loss as effectively as possible. In detail, we expect an improvement in glucose tolerance, insulin sensitivity, and, possibly, beta-cell function, and a decrease in postprandial and daily mean glucose levels. Moreover, we expect that participants with impaired fasting glucose or impaired glucose tolerance will improve their glycemic status more effectively than people with normal glucose tolerance. We also expect a decline in blood pressure, blood triglycerides, total and LDL cholesterol, and possibly in sleep quality upon both eTRE and lTRE interventions. Furthermore, based on previous observations concerning meal timing and TRE, we hypothesize that the restriction of food intake at the beginning of the day (eTRE) will show more beneficial metabolic effects compared with the food intake restriction at the end of the day (lTRE), which might be associated with different molecular signatures in the fasting adipose tissue. We expect that genes and pathways involved in glucose and lipid metabolism, inflammation, oxidative stress, and autophagy, as well as clock genes, will be affected by meal timing. Finally, we suppose that eTRE vs. lTRE will shift the internal circadian time assessed by the BodyTime assay in opposite directions.

## Discussion

ChronoFast is the first randomized clinical trial in Germany directly comparing the cardiometabolic effects of eTRE vs. lTRE in a cohort of overweight and obese woman using a cross-over design. The study is based on previous research on metabolic effects of TRE interventions performed in different cohorts of metabolically healthy or ill subjects (41, 47–64). However, the protocol of the ChronoFast trial considerably extends previous study protocols using novel digital tools of chrononutritional monitoring (electronic food diary, 24 h CGM, and actigraphy) and by including a larger range of cardiometabolic and behavioral outcomes (glucose and lipid levels, metabolic hormones, adipokines and inflammatory biomarkers, blood pressure, satiety/hunger scores, sleep quality, social and economic decision behaviors, and gene expression in adipose tissue and blood cells). These tools and measures will advance the understanding of TRE-induced effects and their mechanisms compared with previous studies.

The most important strength of the ChronoFast study protocol is the careful control of timely compliance, dietary composition, calorie intake, and physical activity during the study. The aim is to achieve as minimal as possible calorie deficit or even stable body weight. As mentioned above, it is unknown whether beneficial metabolic effects of TRE result from the shortening of eating window or from the weight loss that accompanied most published TRE trials (41, 47–59). Nevertheless, four carefully controlled or short-term (4–5 days) TRE trials revealed beneficial metabolic effects without caloric restriction or weight loss (59–61, 81), suggesting that timing factor alone can improve metabolic state. Because study subjects tend to restrict caloric intake upon TRE, several tools will be used in the ChronoFast study to avoid weight loss during the intervention as established previously (21). First, participants will be informed about the importance of maintaining constant body weight for the whole duration of the study. Second, subjects will receive their individual food records and daily caloric intakes made in the run-in phase for guidance. Participants will be asked to document their food selection, consumed amount, and time of each meal daily. Third, they will be instructed to record their weight daily and report any weight fluctuation of ≥ 700 g on 2 consecutive days. The dietician will be trained to counteract weight losses quickly through changes in dietary plans. The dietician will contact study participants by telephone calls in the middle of each intervention and the run-in phase (7 days after visiting the examination center) to increase adherence to the assigned program and answer questions. The same tools will be used to ensure that the macronutrient distribution remains unchanged throughout the study, so that no change in dietary composition will interfere with the limitation of the eating time window or with different eating times. Furthermore, the documentation of the time of each meal, in combination with the monitoring of postprandial glucose excursions by CGM, will ensure the careful control of timely compliance during the intervention. Finally, physical activity will be monitored throughout whole intervention periods by actigraphy. Hence, we will be able to exclude that the observed effects of TRE were influenced by changes in physical activity behavior.

The cross-over design is another very essential strength of the ChronoFast trial protocol, which will allow for the comparison of cardiometabolic effects of eTRE vs. lTRE directly in the same study subjects. Until now, only one cross-over trial that compared the effects of eTRE and lTRE has been published (58), which was accompanied by manifested weight loss after both interventions and, possibly due to this reason, observed no difference in the improvement of glucose and lipid levels between two dietary patterns. Notably, the 10 week study in the United Kingdom, which also aims to compare the metabolic effects of eTRE and lTRE on adults with increased type 2 diabetes risk, is currently ongoing (82). However, this study uses a three-arm parallel study design, whereas the ChronoFast study has a cross-over design, which removes variability between participants because each study subject acts as his own control.

Notably, a range of published TRE studies was conducted only with male participants, especially when time frames are short, as female participants tend to show cyclic changes in body weight and behavior owing to the female cycle. Thus, working with solely female participants is also a strength of this study. Because of the 4 week run-in phase and the detailed recording of baseline characteristics before each intervention, confounding factors will be eliminated.

The last objective of the ChronoFast study is assessment and comparison of molecular mechanisms induced by eTRE and lTRE in adipose tissue and blood monocytes. Numerous molecular mechanisms underlying the metabolic effects of TRE are intensively investigated in animal studies (45, 46, 83). Postprandial increase in various nutrients, such as glucose, lipids, and amino acids, affects both glucose and lipid metabolism and circadian clock pathways *via* key intracellular metabolic sensors such as SIRT1, mTOR, S6K, AMPK, PPARs, RORs, and Rev-Erbs (84). Notably, insulin (and possibly other meal-induced hormones) can directly entrain the circadian clock in liver and adipose tissue and in this way additionally affect metabolic outcomes (85, 86). The elegant study of Mukherji et al. (83) confirmed a central role of PPARα, Rev-Erbα, and CREB in circadian and metabolic changes induced by shifting of feeding time. However, most of these mechanisms were described in rodents and require intensive investigation in clinical settings to prove feasibility, safety, and effectiveness.

As mentioned above, we expect that genes and pathways involved in glucose and lipid metabolism, inflammation, oxidative stress, and autophagy, as well as clock genes, will be affected by meal timing, so significant differences will be observed between eTRE and lTRE. However, and this is a main limitation of the trial, most data will only be collected at one circadian time point, i.e., in the morning after the overnight fast. Hence, these transcriptome data will represent a one-moment molecular signature in the fasting adipose tissue and cannot validate circadian transcriptional response to altered feeding schedules observed in rodents, for which multiple sampling over 24 h [or at least at several time points (73)] is necessarily. Indeed, for circadian regulated genes, expression changes observed at one circadian time point will not necessarily mean that there is improvement or worsening. Because food intake is a potent *zeitgeber*, observed gene expression change may be a result of a phase shift in the rhythmicity meaning that if the same test is done later in the day, there will be no differences or changes in the opposite direction. Nevertheless, the ChronoFast study will provide novel data on the TRE-dependent regulation of non-circadian transcriptome (which is expectedly a larger part of adipose tissue transcriptome) and, in this way, expand the very scarce knowledge of the regulation of adipose tissue by meal timing in humans. Interestingly, a recent study investigating subcutaneous adipose tissue samples taken at 6 hourly intervals over 24 h under highly controlled “constant routine” conditions, observed only ~2% transcripts exhibiting circadian expression profiles (87), although other tissues showed that 10–30% transcriptome is circadian. However, if the expression of known circadian output genes is changed, the transcriptome data have to be interpreted with caution.

The same limitation has to be considered for the analysis of strongly circadian regulated clinical outcomes. However, most metabolic measures planned in the protocol (such as glucose, insulin, and incretins) are relatively stable in the fasting state and are much stronger affected by the meal intake than by the endogenous circadian rhythm. Such endogenous circadian rhythms usually show a relatively small amplitude and could be observed mostly in carefully controlled constant routine experiments. Therefore, it cannot be expected that the timing of food intake on the day before will meaningfully affect metabolic parameters in the morning after the overnight fast because of the phase shift in rhythmicity.

## Conclusions

The protocol of the ChronoFast clinical trial was designed to determine whether eTRE or lTRE is a more effective dietary approach for the improvement of glycemic control, lipid metabolism, inflammation, and sleep quality in overweight and obese women who have an increased risk of type 2 diabetes. The hallmarks of the study will be (1) the homogenous group of female study subjects, (2) randomized cross-over study design, (3) carefully monitored food intake combined with extensive nutritional counseling that will ensure timely compliance, unchanged dietary composition, and as minimal as possible calorie deficit, (4) carefully monitored physical activity, and (5) extensive investigation of molecular mechanisms of TRE in adipose tissue and blood monocytes.

The obtained data may contribute to the development of a simple and cost-effective dietary approach for the prevention and therapy of glucose metabolism disturbances in subjects with obesity and in the general population. We expect that our study will lay foundations to confirm our findings in future large-scale and/or large-term TRE studies and in diverse subject groups (e.g., subjects of different age, gender, and chronotypes and subjects with diabetes, metabolic syndrome, etc.).

## Data Availability Statement

The original contributions presented in the study are included in the article/supplementary material, further inquiries can be directed to the corresponding authors.

## Ethics Statement

The studies involving human participants were reviewed and approved by Ethics Committee of the University of Potsdam (application No. 8/2019, approved on 25.10.2019). The patients/participants provided their written informed consent to participate in this study.

## Author Contributions

OP-R, DK-L, AP, AK, and AM: conceptualization. OP-R, NS, DK-L, and AK: methodology. NS: software. BP, BS, and OP-R: validation and formal analysis. BP and BS: investigation and data curation. OP-R and AK: resources. BP and OP-R: writing—original draft preparation. AP, AK, DK-L, and AM: writing—review and editing. OP-R: visualization, supervision, project administration, and funding acquisition. All the authors have read and approved the final manuscript.

## Funding

The study is funded by the German Science Foundation (DFG RA 3340/3-1, OP-R, by the German Diabetic Association (DDG, Allgemeine Projektförderung, 2020, OP-R; Adam Heller Funding provided by DDG/Abbott, 2021, OP-R), and by the European Association of Study of Diabetes (Morgagni Prize, 2020, OP-R). Funders are not involved in the study design, collection, analysis, interpretation of data, writing of this article, or the decision to submit it for publication.

## Conflict of Interest

The authors declare that the research was conducted in the absence of any commercial or financial relationships that could be construed as a potential conflict of interest.

## Publisher's Note

All claims expressed in this article are solely those of the authors and do not necessarily represent those of their affiliated organizations, or those of the publisher, the editors and the reviewers. Any product that may be evaluated in this article, or claim that may be made by its manufacturer, is not guaranteed or endorsed by the publisher.
